# Association between Circulating B-Type Natriuretic Peptide and Diabetic Peripheral Neuropathy: A Cross-Sectional Study of a Chinese Type 2 Diabetic Population

**DOI:** 10.1155/2020/3436549

**Published:** 2020-10-12

**Authors:** Pijun Yan, Qin Wan, Zhihong Zhang, Yong Xu, Ying Miao, Pan Chen, Chenlin Gao

**Affiliations:** ^1^Department of Endocrinology, The Affiliated Hospital of Southwest Medical University, Luzhou, Sichuan 646000, China; ^2^Department of General Medicine, The Affiliated Hospital of Southwest Medical University, Luzhou, Sichuan 646000, China

## Abstract

Cardiovascular disease which is associated with cardiac dysfunction, usually measured with circulating levels of B-type natriuretic peptide (BNP), has been associated with incidence and progression of diabetic peripheral neuropathy (DPN). The potential relationship of circulating physiological levels of BNP with DPN, however, has not been reported. Circulating levels of BNP were measured in 258 patients with type 2 diabetes mellitus (T2DM), and participants were divided into a DPN group (*n* = 61) and no DPN group (*n* = 197). The relationship between circulating physiological levels of BNP and DPN and other parameters was analyzed. Circulating levels of BNP were significantly elevated in T2DM patients with DPN compared to those without (*P* = 0.001). Circulating levels of BNP were significantly and positively associated with systolic blood pressure (*P* = 0.035), neutrophil-to-lymphocyte ratio (*P* = 0.007), creatinine (*P* = 0.030), vibration perception threshold values (*P* = 0.021), and the prevalence of diabetic foot ulceration (*P* = 0.039), peripheral arterial disease (*P* = 0.013), DPN (*P* = 0.032), and diabetic nephropathy (*P* = 0.020) and negatively with lymphocyte count (*P* = 0.003) and ankle-brachial index (*P* = 0.038), irrespective of age, sex, and body mass index. Moreover, circulating levels of BNP was an independent decisive factor for the presence of DPN after multivariate adjustment (odds ratio, 1.044; 95% confidence interval, 1.006-1.084; *P* = 0.024). Additionally, the higher quartiles of circulating BNP were related significantly to an increased risk of DPN compared to the lowest quartile (*P* = 0.003). Last but most importantly, the analysis of receiver operating characteristic curves revealed that the best cutoff value for circulating levels of BNP to predict DPN was 15.18 pg/mL (sensitivity 78.7% and specificity 48.2%). These findings suggest that high circulating physiological levels of BNP may be associated with the development of DPN and may be a potential biomarker for DPN in patients with T2DM.

## 1. Introduction

Diabetic peripheral neuropathy (DPN) is the most common form of neuropathy among diabetic patients with complex, multifactorial pathogenetic mechanisms, resulting in peripheral nerve dysfunction accompanied with typical characteristics of pain and numbness [1]. DPN has been regarded as a major risk factor for foot infection, ulcers, and subsequent amputations, which result in a heavy economic burden, and become an alarming global health issue. Currently, apart from intensive glucose control, there is no approved therapy to prevent or cure DPN [1]. Thus, it is of great clinical importance that identifying novel risk factors for DPN may help in the development of appropriate strategies for the prevention and treatment of DPN in patients with type 2 diabetes mellitus (T2DM).

B-type natriuretic peptide (BNP), a 32-amino acid neurohormone produced in and secreted from the heart in response to ventricular dilatation and pressure overload and ischemic injury, has been shown to predict cardiovascular disease (CVD) outcomes in different populations and widely used as a diagnostic and prognostic biomarker for heart failure in clinical practice [2, 3]. It has also been reported to early reflect left ventricular systolic and diastolic dysfunction, while subclinical left ventricular dysfunction is related to the dysfunction of the adrenergic cardiac innervation [4] and implicated in the development of cardiovascular autonomic neuropathy (CAN), one of the main types of diabetic neuropathy, and DPN [5], indirectly indicating that BNP might be associated with the pathogenesis of DPN. There is increasing evidence that BNP can mediate peripheral vasodilation and exert antifibrosis, anti-inflammatory, and antioxidative stress effects [2, 3], and altered levels of circulating BNP have been reported to contribute to other diabetic microangiopathy such as diabetic nephropathy (DN), diabetic retinopathy (DR), and CAN [2, 6, 7]. It is well known that diabetic microangiopathy is closely interconnected and has shared pathogenetic mechanisms. Therefore, these results indicate that there may exist a potential association between circulating BNP and the development of DPN. Consistently, two studies performed by Jurado et al. and Hamano et al. have reported a significant positive relationship between levels of N-terminal pro-BNP (NT-proBNP), a biologically inactive fragment of BNP, and the presence of DPN, independently of previous CVD or cardiovascular risk factors such as body mass index (BMI) and glycated hemoglobin A1C (HbA1c), in patients with T2DM [6, 7]. Similar findings were observed in patients with type 1 diabetes [8]. Collectively, these data suggest that circulating BNP levels may be associated with the development of DPN, but the mechanisms underlying this association remain poorly understood. Moreover, no study has explored whether circulating physiological levels of BNP may serve as a risk marker for DPN and to what extent it is associated with DPN.

Therefore, The present study was designed to compare circulating physiological levels of BNP, cardiometabolic parameters, inflammatory marker, and other vascular complications in a Chinese population of T2DM patients with preserved cardiac function, with and without DPN, and assess their contribution to DPN in such patients.

## 2. Materials and Methods

### 2.1. Study Population

All T2DM patients were recruited from our inpatient department between August 2012 and September 2015. A total of 258 T2DM patients aged 25–89 years, with circulating levels of BNP ≤ 100 pg/mL, were eligible. All subjects completed a standard questionnaire including diabetic duration, lifestyle habits (alcohol consumption and cigarette smoking), previous or current diseases (hypertension, stroke, coronary heart disease (CHD), diabetic foot ulceration (DFU), peripheral arterial disease (PAD), DR, DN, and other diseases), and use of medications and underwent a comprehensive physical examination according to standard procedures. The exclusion criteria were endocrine disorders other than T2DM, acute complications of diabetes, evidence of any amputation, lower limb elective angioplasty or stent placement, any chronic neurological diseases such as Parkinson's disease and multiple sclerosis, history of seizures or epilepsy, cardiac arrhythmias such as atrial fibrillation, aortic stenosis, uncontrolled hypertension > 180/100 mmHg, symptomatic heart failure (New York Heart Association (NYHA) classes II–IV), acute respiratory failure or severe obstructive lung disease, severe renal failure defined as estimated glomerular filtration rate (eGFR) < 30 mL/min/1.73 m^2^, chronic liver disease, thromboembolic disease, hematological system diseases, inflammatory or autoimmune diseases, infectious disease, alcoholism, malignancies, pregnancy or lactation, any other etiological cause of peripheral neuropathy (e.g., peripheral polyneuritis, infectious polyneuritis, vasculitis, and cervical and lumbar spondylosis), and use of immunosuppressant, anti-inflammatory agents, analgesics, diuretic, systemic corticosteroids, and neurotoxic agents. 258 eligible participants were subsequently divided into two groups (DPN and non-DPN group) according to a previous published work.

The study was performed in accordance with the ethical guidelines of the 1975 Declaration of Helsinki and was reviewed and approved by the human research ethics committee of the Affiliated Hospital of Southwest Medical University, and informed consent was obtained from all T2DM patients prior to participation.

### 2.2. Anthropometric and Biochemical Measurements

Body weight and height were measured with the subject wearing light clothes without shoes, and BMI was then calculated. Blood pressure was measured on the right arm using a standard mercury sphygmomanometer in the supine position after 10 min of rest.

Blood samples were collected from study participants after at least 8 h of fasting to measure fasting blood glucose (FBG); HbA1c; albumin; lipid profiles, including total cholesterol (TC), triglyceride (TG), high-density lipoprotein cholesterol (HDL-C), and low-density lipoprotein cholesterol (LDL-C); serum creatinine; white blood cell (WBC); neutrophil; lymphocyte counts; neutrophil-to-lymphocyte ratio (NLR); coagulation function tests, including prothrombin time (PT) and activated partial thromboplastin time (APTT); international normalized ratio (INR), and fibrinogen; and circulating BNP. The circulating level of BNP was quantified using an immunochemiluminometric assay according to the manufacturer's instruction, and its normal range used in the study was 0–100 pg/mL. All tests used in this study were conducted according to relevant protocols and guidelines at a certified laboratory. Urinary microalbumin and creatinine were measured, and the urinary albumin-to-creatinine ratio (ACR; mg/g creatinine) was calculated, as we described previously [9]. Renal function expressed as the eGFR was calculated using Chronic Kidney Disease Epidemiology Collaboration (CKD-EPI) equations modified by a Japanese coefficient [9]. Patients were then classified as having DN if they had an eGFR < 60 mL/min/1.73m^2^ and/or an ACR > 30 mg/g [9, 10].

Ankle-brachial index (ABI) measurements were measured by a continuous-wave Doppler ultrasound probe (Vista AVS, Summit Co.) in all T2DM patients. PAD was defined as ABI < 0.90 [9, 11].

### 2.3. Diagnostic Criteria of DPN

Vibration perception thresholds (VPT) was assessed at the metatarsophalangeal joint dig I using a neurothesiometer (Bio-Thesiometer; Bio-Medical Instrument Co., Newbury, OH, USA) according to previously published methods. Sensibility to touch was tested using 10 g Semmes-Weinstein monofilament at four points on each foot: three on the plantar and one on the dorsal side. The same experienced physician performed all the above measurements in a quiet, warm, relaxed environment. DPN was defined as VPT ≥ 25 V and/or inability to feel the monofilament [1, 9].

### 2.4. Statistical Analysis

All analyses were performed with the Statistical Package for Social Sciences version 20.0 (SPSS, Chicago, IL). All data were first analyzed for normality of distribution using the Kolmogorov-Smirnov test and homogeneity of variance using the Levene homogeneity of variance test. Data are expressed as mean ± standard deviation (SD) for continuous variables or number (percentages) for categorical variables.

Differences between participants with and without DPN were assessed using the *χ*^2^ test for categorical variables and Student's *t*-test or Mann–Whitney *U* test for continuous variables, when appropriate. Correlation analysis was used to evaluate the relationship between circulating levels of BNP and other variables; the partial correlation coefficient was used to control for the effects of age, gender, and BMI. The univariate and multivariable logistic regression analyses were also performed to determine the association of circulating levels of BNP and other variables with risk of DPN. We then categorized patients into four quartile groups by circulating levels of BNP level. Binary logistic regression analyses were conducted to investigate the association between quartiles of circulating BNP and DPN. The lowest quartile (Q1) served as the reference group. Odds ratios (ORs) and 95% confidence intervals (CI) were estimated. Possible dose-response relationships between circulating levels of BNP and DPN were examined by the trend test. Last, receiver operating characteristic (ROC) curve analysis was performed to determine the optimal cutoff point of circulating levels of BNP for the diagnosis of DPN.

In all statistical tests, a *P* value of <0.05 was considered to be statistically significant (two sided).

For sample size calculation, we used the following formula:

(1) $$\begin{array}{c} N=\frac{\left(q_{1}^{-1}+q_{2}^{-1}\right){\left(t_{\alpha /2}+t_{\beta }\right)}^{2}S^{2}}{\delta^{2}} \end{array}$$

where *N* is the total sample size (the sum of the sizes of both comparison groups). In our study, it is assumed that test level *α* = 0.05 and *β* = 0.1, and the population mean difference between the T2DM patients without DPN group and the T2DM patients with DPN group is 3.5 (*δ* = 3.5). Also, we assume that the sample size ratio between the T2DM patients with DPN group and the T2DM patients without DPN group is 1 : 3.5. Accordingly, *q*_1_ (T2DM patients with DPN group) = 1/(1 + 3.5) = 0.22, and *q*_2_ (T2DM patients without DPN group) = = 3.5/(1 + 3) = 0.78. A previous work has proposed that the SD of circulating BNP for the T2DM patients without DPN group is 7 (*S* = 7) [7, 12]. Then, all parameters are included in the above formula, and we get the following result:

(2) $$\begin{array}{c} N=\frac{\left[{\left(0.22\right)}^{-1}+{\left(0.78\right)}^{-1}\right]{\left(1.960+1.282\right)}^{2}\times {\left(7\right)}^{2}}{3.5^{2}}=246.35\approx 247 \end{array}$$

From the result, we have known that the minimum sample size in the T2DM patients without DPN group is 193, and the minimum sample size in the T2DM patients with DPN group is 55. In other words, this study has a test efficacy of 0.90 at a two-sided *α* of 0.05 as long as the sample size in the T2DM patients without DPN group is ≥193 and the sample size in the T2DM patients with DPN group is ≥55.

## 3. Results

### 3.1. Circulating Levels of BNP and Other Clinical Characteristics of Studied Population

A total of 258 patients with T2DM (mean age, 63.77 ± 10.76 years; male/female, 92/166; and mean diabetes duration, 8.68 ± 6.78 years) were finally enrolled in this study. The anthropometric, biochemical, and clinical parameters of studied population are shown in Table 1. When compared with those without, T2DM patients with DPN had significantly older age (*P* = 0.014); higher BNP (*P* = 0.001), neutrophil count (*P* = 0.026), NLR (*P* < 0.001), fibrinogen (*P* = 0.023), ACR (*P* = 0.004), creatinine (*P =0.007*), and VPT (*P* < 0.001); larger proportions of DFU (*P* = 0.002), PAD (*P* = 0.001), DN (*P* < 0.001), and DR (*P* = 0.032); and lower BMI (*P* < 0.001), eGFR (*P* = 0.002), lymphocyte count (*P* < 0.001), and ABI (*P* = 0.028).

### 3.2. Association of Circulating Levels of BNP with Anthropometric, Biochemical, and Clinical Parameters in Study Subjects

Next, we analyzed the relationship of circulating levels of BNP with various other parameters by using simple correlations. In all T2DM patients, circulating levels of BNP were positively associated with sex (*P* = 0.010), age (*P* < 0.001), diabetic duration (*P* < 0.001), SBP (*P* < 0.001), HDL-C (*P* = 0.024), NLR (*P* = 0.015), urinary ACR (*P* < 0.001), VPT values (*P* = 0.002), and the prevalence of hypertension (*P* = 0.002), CHD (*P* = 0.001), stroke (*P* = 0.039), PAD (*P* < 0.001), DPN (*P* = 0.001), and DN (*P* = 0.001) and negatively with TG (*P* = 0.004), lymphocyte count (*P* = 0.001), eGFR (*P* < 0.001), and ABI (*P* = 0.038) (Table 2). With adjustment for age, sex, and BMI, circulating levels of BNP were correlated significantly and positively with SBP (*P* = 0.035), NLR (*P* = 0.007), creatinine (*P* = 0.030), VPT values (*P* =0.021), and the prevalence of DFU (*P* = 0.039), PAD (*P* = 0.013), DPN (*P* = 0.032), and DN (*P* = 0.020) and negatively with lymphocyte count (*P* = 0.003) and ABI (*P* = 0.038) (Table 2).

### 3.3. Multivariable-Adjusted ORs for the Association of Circulating Levels of BNP with Increased Presence of DPN in Study Subjects

To assess whether circulating levels of BNP can decrease the risk of development of DPN, univariate and multivariate logistic regression analysis was mapped. As shown in Table 3, univariate logistic regression analysis revealed that BMI, lymphocyte count, and eGFR were negative predictors of the presence of DPN, and age, diabetic duration, FBG, HbA1c, neutrophil count, NLR, fibrinogen, creatinine, BNP, and the prevalence of DR, PAD, and DFU were positive predictors of the presence of DPN. Importantly, the circulating level of BNP remained independently significantly associated with the presence of DPN when assessed in a multiple logistic regression model (odds ratio, 1.044; 95% confidence interval, 1.006-1.084; *P* = 0.024), indicating that there was a 4.4% increase in the odds of having DPN for each 1 pg/mL increase in circulating levels of BNP.

### 3.4. Association between Quartiles of Circulating Levels of BNP and the Risk of DPN in Study Subjects

Further, all subjects were separately categorized into four quartile groups (Q1–Q4) according to circulating levels of BNP, and the risk of development of DPN in different BNP quartiles was assessed. As shown in Table 4, there was a 49.4% increase in DPN risk per SD increase in BNP (OR = 1.494, 95% CI 1.130-1.963). The ORs for DPN were progressively increased with an increasing BNP quartiles (*P* for trend = 0.008). Compared with BNP Q1 (referent), patients in Q2 (OR, 2.900; 95% CI, 1.046-8.038; *P* < 0.05), Q3 (OR, 5.294; 95% CI, 1.982-14.138; *P* < 0.01), and Q4 (OR, 3.496; 95% CI, 1.277-9.572; *P* < 0.05) had significantly higher risk of DPN.

### 3.5. The Predictive Value of Circulating Levels of BNP in Detecting DPN

**Figure 1:**
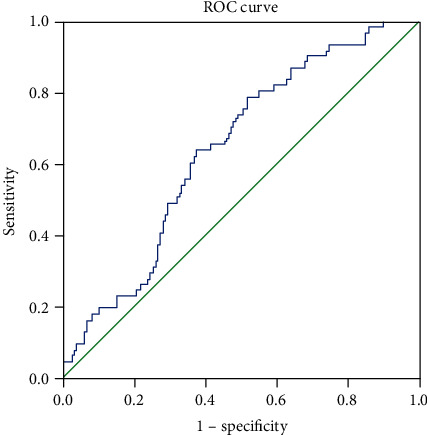
ROC analysis of circulating levels of B-type natriuretic peptide (BNP) to indicate DPN for T2DM patients. AUC = 0.643; 95% CI, 0.569–0.716; *P* < 0.001; identified BNP cutoff value = 15.18 pg/mL; Youden index = 0.269; sensitivity: 78.7%; specificity: 48.2%.

To explore the predictive value of circulating levels of BNP for DPN, we analyzed the ROC curves of circulating levels of BNP. The results revealed that the best cutoff value for circulating levels of BNP to predict DPN was 15.18 pg/mL (sensitivity: 78.7%, specificity: 48.2%, and AUC 0.643) in patients with T2DM (Figure 1).

## 4. Discussion

To date, this was the first study to explore the relationship between circulating physiological levels of BNP and the risk of DPN. We found that circulating levels of BNP significantly increased in T2DM patients with DPN and were independently and positively associated with VPT values. We further showed that the circulating level of BNP was an independent decisive factor for the presence of DPN after multivariate adjustment and circulating BNP = 15.18 pg/mL was found to predict the presence of DPN. These findings suggest that circulating BNP may be a useful biomarker of DPN, and high physiological circulating BNP concentrations may be associated with increased risk of DPN.

BNP and its inactive cleavage product NT-proBNP are predominantly synthesized at equimolar levels by the ventricle myocytes and released into the circulation in response to ventricular dilatation, pressure overload, or myocardial ischemia [2, 3]. A cross-sectional pilot study of 104 T2DM patients performed by Jurado and coworkers demonstrated that T2DM patients with DPN disclosed significantly higher serum NT-proBNP, and serum NT-proBNP was significantly associated with DPN independently of previous CVD [6]. Similarly, Hamano et al. reported that the OR of having DPN was 6.6 for the highest quartile of the NT-proBNP level compared with the lowest quartile level after adjustments for age, sex, diabetes duration, BMI, and HbA1c [7]. Consistent with these results reported by Jurado et al. and Hamano et al. [6, 7], we found that T2DM patients with DPN had significantly higher circulating levels of BNP compared with those without, and circulating levels of BNP were positively associated with VPT, an indicator of confirmed clinical neuropathy. This suggests that there may be a potential mechanistic association between higher circulating BNP and the development of DPN. Moreover, multivariate logistic regression analysis revealed that the circulating level of BNP was an independent decisive factor for the presence of DPN after multivariate adjustment. Additionally, per SD, increase in BNP was associated with an increase in the prevalence of DPN by 49.4% (95% CI: 13.0–96.3%). Compared with participants who were categorized to the lowest quartile of BNP level (Q1), those with a higher quartile of BNP level (Q2, Q3, and Q4) showed a 2.9-, 5.3-, and 3.5-fold increase in the prevalence of DPN, respectively. These data consistently demonstrated that a high level of circulating BNP may be an independent risk factor of DPN, the level of circulating BNP is higher, and the risk of DPN in patients with T2DM is greater. Last but more importantly, circulating BNP was found to predict the presence of DPN. Both our hereby presented findings and the results of previous studies indicate that higher physiological levels of circulating BNP may be associated with the development of DPN, and circulating BNP might be a potential biomarker for DPN in patients with T2DM.

Chronic hyperglycemia is considered to play a crucial role in the development and progression of DPN. It has been reported that chronic hyperglycemia stimulates the formation of advanced glycated end products (AGEs) and their receptors, causes the overproduction of nicotinamide adenine dinucleotide, and leads to activation of poly (ADP-ribose) polymerase, hexosamine, and protein kinase C pathways, aggravating oxidative stress generation, subsequently activating inflammatory responses and neuronal dysfunction, and eventually leading to the onset and progression of diabetic neuropathy [13–15]. Several studies have demonstrated that poor glycemic control contributes to the development of diabetic neuropathy, whereas good control improves vibratory sensitivity and nerve conduction velocity [16, 17]. HbA1c represents blood glucose control conditions for recent 8-12 weeks, and the state of glycemic control was evaluated from FBG and HbA1C in the present study. We found that T2DM patients with DPN had slightly but not significantly higher FBG and HbA1c when compared with those without DPN. This might be a result of most of the patients in our study having been admitted to the hospital to treat severely poor glycemic control, and the long-term control of blood glucose might not have been reflected through the levels of HbA1c and FBG detected at admission. In addition, this might be also result of previous and recent therapy. As expected, the univariate logistic regression analysis in our study revealed that FBG and HbA1c were positive predictors of the presence of DPN, and multivariate logistic regression analysis revealed that HbA1c was an independent decisive factor for the presence of DPN, consistent with previous studies demonstrating that HbA1c has been shown to be related to the incidence and the prevalence of diabetic DSP in both cross-sectional and prospective epidemiological studies [18–20]. Correlation analysis showed that circulating levels of BNP were not associated with FBG and HbA1c in patients with T2DM before and after adjustment for age, gender, and BMI. Moreover, multivariate logistic regression analysis revealed that circulating BNP remained significantly associated with the presence of DPN independently of FBG and HbA1c. These results indicate that poor glycemic control may not mediate the potential association of circulating BNP and DPN; however, these findings warrant further investigation.

Experimental and epidemiological studies have shown that chronic low-grade inflammation and activation of the innate immune system play a critical role in the development and progression of DPN [2, 21]. NLR, a novel and useful biomarker of systemic inflammation in clinical practice, has high value because it integrates information that derives from two immune pathways, the neutrophils, responsible for ongoing inflammation, and the lymphocytes that represent the regulatory pathway [22]. Our study provides further evidence that supports the potential role of a dysregulated inflammatory response in the development of DPN, since we found that T2DM patients with DPN had significantly increased inflammatory markers (neutrophil count, NLR, and fibrinogen) and decreased lymphocyte count, and these inflammatory markers were all predictors of the presence of DPN. Further, our data demonstrated that circulating levels of BNP were correlated significantly and positively with NLR and negatively with lymphocyte count. In accordance with our data, Avci et al. found that NLR was positively associated with NYHA functional class and plasma BNP and negatively with left ventricular ejection fraction, the most commonly used clinical measure of left ventricular systolic function and a good indicator of cardiac remodeling [23], and NLR was an independent predictor of increased levels of plasma BNP in patients with idiopathic dilated cardiomyopathy [24]. Altogether, these findings strongly suggest that circulating levels of BNP may be associated with inflammation, and inflammation might mediate the potential association of the circulating level of BNP and DPN. Clinical studies suggest that natriuretic peptide levels are, at least partly, elevated in response to either increased secretion or decreased degradation due to inflammation [25]. Animal studies and tissue cultures also show that the production and the secretion of natriuretic peptides are activated by endotoxin and inflammatory mediators [25]. An experimental in vitro study also reported that lipopolysaccharides and proinflammatory cytokines could stimulate the BNP mRNA expression and protein secretion dose-dependently in cultured rat myocytes via p38 MAP kinase activation [26]. Recently, it has been demonstrated that BNP can exert anti-inflammatory effects including suppression of neutrophil superoxide (O_2_(-)) generation and release by suppressing NAD(P)H oxidase assembly [27]. These findings revealed that an elevated circulating level of BNP is linked to inflammation in T2DM patients with DPN, and increase in circulating BNP as a compensatory mechanism may exert beneficial effects on repair of the injured neurons by modulating neuroinflammatory responses [28]; further studies are needed to fully elucidate its mechanism of action.

Growing evidence suggests that vascular complications associated with atherosclerosis in patients with T2DM are a critical element to the pathogenesis of DPN [1]. It is well known that ABI is a noninvasive diagnostic biomarker for lower-extremity PAD and also a reliable indicator of atherosclerosis at other vascular sites [29]. Microalbuminuria has been recognized for a long time as an indicator of endothelial dysfunction and early DN [30] and a strong predictor of future progression of renal dysfunction and cardiovascular events in T2DM patients [2, 7, 30]. Our study findings provided further evidence that T2DM patients with DPN had significantly larger proportions of other diabetic vascular complications, including DFU, PAD (lower ABI), DN (higher urinary ACR and serum creatinine and lower eGFR), and DR, and, meanwhile, the prevalence of DR, PAD, and DFU and lower eGFR were positive predictors of the presence of DPN, consistent with previous studies [10, 31, 32], suggesting that diabetic vascular complications are closely interconnected, and nerve ischemia associated with vascular complications may contribute to nerve damage, eventually leading to the development of DPN [2, 33, 34]. We further demonstrated that circulating levels of BNP were correlated significantly and positively with SBP and the prevalence of DFU, PAD, and DN and negatively with ABI, in agreement with previous studies [1, 30, 35, 36]. It is well known that blood pressure and renal function are important factors that may regulate the plasma concentration of natriuretic peptides, and patients with hypertension and DN had elevated plasma levels of BNP [7, 2, 36], and NT-proBNP levels were positively associated with blood pressure levels, especially SBP [2, 28, 30, 36]. In addition, animal studies have shown that chronic excess of BNP in mice prevents diabetic glomerular injury, with amelioration of albuminuria and renal dysfunction [37], implying a renoprotective effect of circulating BNP. Several studies have shown that circulating levels of BNP and NT-proBNP are markedly elevated in patients with PAD, and the BNP level was negatively correlated with ABI [7, 38, 39]. Transgenic mice that overexpress BNP in response to hind limb ischemia have accelerated vascular regeneration following experimental femoral artery ligation [40]. Recent studies suggest that BNP is an independent predictor of increased risk of secondary interventions or limb loss in patients with PAD [41]. Together, these lines of evidence, combined with the results of our present study, suggest that functional abnormalities in the micro- and macrovasculature can lead to ischemia, which would induce BNP synthesis via wall motion abnormalities and an increase in wall stress, or that ischemia can directly promote the release of BNP in a manner independent from cardiomyocytes, and subsequently, possible rises in BNP of circulating levels [34, 42] and compensatory increase in circulating BNP may exert neuroprotective effect in T2DM patients with DPN by modulating neuroinflammatory response, promoting angiogenesis, modifying vascular endothelial function, reducing cardiac load, and improving blood supply to nerve fibers by diuretic and vasodilatory effects [38]. However, because of the impairment of BNP receptors in atherosclerosis or ischemic vascular disease, the protective effect of BNP is weakened [39] and the specific mechanism remains to be elucidated.

Some potential limitations of our study should be noted. First, the single-center cross-sectional design precluded conclusions on the temporal relationship between elevated levels of circulating BNP and DPN. Thus, larger prospective and randomized studies raising circulating BNP would be better suited to establishing the causal link. Second, the enrolled subjects were all inpatients with T2DM, who generally had more serious illness than diabetic outpatients, and thus, the generalizability of our findings to differing populations cannot be assumed. Third, we only evaluated fibrinogen and leukocytes and its subtypes as inflammation markers. The lack of classical inflammatory markers such as high-sensitivity C-reactive protein, interleukin-6, and tumor necrosis factor-*α* or eotaxin analysis and/or correlation makes it difficult to draw any consistent conclusion regarding the possibility of leukocytes and its subtypes to predict evaluated circulating BNP and DPN. Fourth, as we did not obtain measurement parameters, such as left ventricular ejection fraction, left ventricular posterior wall thickness, and interventricular septal thickness, detected with Doppler echocardiogram in all participants that thus were not analyzed in the final results, which might have influenced the results. Despite these limitations, the current study is not without strengths, some of which are relatively large sample size, use of a standardized method at a single center, and thorough adjustment for possible confounding variables, which can raise the reliability of our findings. Moreover, our study is, to our knowledge, the first to evaluate the association between circulating physiological levels of DPN in Chinese inpatients with T2DM and may provide a possible mechanism for circulating BNP in the pathogenesis of DPN.

## 5. Conclusions

The present study showed that circulating levels of BNP significantly increased in T2DM patients with DPN, and high physiological level of circulating BNP is an independent predictor of the presence of DPN in Chinese patients with T2DM, thereby suggesting that circulating levels of BNP were significantly associated with the presence of DPN, and circulating BNP may be used as a useful biomarker of DPN risk. However, the underlying pathophysiological mechanisms and clinical implications of these findings warrant further investigation.

## Figures and Tables

**Table 1:** Circulating levels of BNP and other clinical characteristics between T2DM patients with and without DPN.

Variables	No DPN	DPN	*P*
	(*n* = 197)	(*n* = 61)	
Male/female	65/132	27/34	0.109
Age (years)	62.86 ± 10.70	66.70 ± 10.51	0.014
BMI (kg/m^2^)	24.97 ± 3.64	22.90 ± 4.36	<0.001
Diabetic duration (years)	8.19 ± 6.29	10.29 ± 8.03	0.170
SBP (mmHg)	136.06 ± 23.57	133.36 ± 25.92	0.446
DBP (mmHg)	70.37 ± 13.45	69.39 ± 12.57	0.615
FBG (mmol/L)	10.43 ± 4.73	12.31 ± 7.25	0.206
HbA1c (%)	9.51 ± 2.34	10.32 ± 3.17	0.068
TC (mmol/L)	4.86 ± 1.08	4.66 ± 1.20	0.224
TG (mmol/L)	2.25 ± 1.84	1.91 ± 1.44	0.087
HDL-C (mmol/L)	1.19 ± 0.33	1.24 ± 0.36	0.276
LDL-C (mmol/L)	2.79 ± 0.87	2.67 ± 0.87	0.349
Neutrophil count (∗10^9^/L)	4.48 ± 1.56	5.18 ± 2.21	0.026
Lymphocyte count (∗10^9^/L)	1.63 ± 0.58	1.30 ± 0.49	<0.001
NLR	3.19 ± 1.93	4.84 ± 3.87	<0.001
WBC (∗10^9^/L)	6.65 ± 1.71	6.82 ± 2.17	0.517
PT (s)	12.37 ± 1.09	12.55 ± 0.73	0.249
APTT (s)	30.51 ± 4.66	32.09 ± 6.86	0.194
INR	1.02 ± 0.08	1.03 ± 0.07	0.674
Fibrinogen (g/L)	3.50 ± 1.03	4.12 ± 1.51	0.023
BNP (pg/mL)	21.49 ± 18.98	30.22 ± 22.17	0.001
ACR (mg/g)	132.56 ± 35.62	284.74 ± 103.62	0.004
Creatinine (*μ*mol/L)	65.26 ± 24.73	77.24 ± 31.06	0.007
eGFR (mL/min/1.73 m^2^)	91.36 ± 22.25	80.66 ± 24.46	0.002
VPT (V)	14.23 ± 4.56	36.89 ± 8.99	<0.001
ABI	1.01 ± 0.17	0.94 ± 0.24	0.028
Hypertension (*n*, %)	112 (56.85)	39 (63.93)	0.328
CHD (*n*, %)	27 (13.71)	13 (21.31)	0.152
Stroke (*n*, %)	45 (22.84)	20 (32.79)	0.119
DFU (*n*, %)	6 (3.05)	8 (13.11)	0.002
PAD (*n*, %)	23 (11.68)	18 (29.51)	0.001
DN (*n*, %)	66 (33.50)	36 (59.02)	<0.001
DR (*n*, %)	21 (10.66)	13 (21.31)	0.032

Data are mean ± SD. SD: standard deviation; BMI: body mass index; SBP: systolic blood pressure; DBP: diastolic blood pressure; FBG: fasting blood glucose; HbA1c: glycated hemoglobin A1c; TC: total cholesterol; TG: triglyceride; HDL-C: high-density lipoprotein cholesterol; LDL-C: low-density lipoprotein cholesterol; NLR: neutrophil-to-lymphocyte ratio; WBC: white blood cell; RDW: red cell distribution width; PT: prothrombin time; APTT: activated partial thromboplastin time; INR: international normalized ratio; BNP: B-type natriuretic peptide; ACR: albumin-to-creatinine ratio; eGFR: estimated glomerular filtration rate; VPT: vibration perception threshold; ABI: ankle-brachial index; CHD: coronary heart disease; DFU: diabetic foot ulceration; PAD: peripheral arterial disease; DN: diabetic nephropathy; DR: diabetic retinopathy; DPN: diabetic peripheral neuropathy; T2DM: type 2 diabetes mellitus.

**Table 2:** Linear correlation analysis of variables associated with circulating levels of BNP in the entire population studied.

Variable	Simple			
	*r*	*P* value	Adjusted *r*	Adjusted *P* value
Age	0.363	<0.001	—	—
Sex	0.160	0.010	—	—
BMI	-0.024	0.711	—	—
Diabetic duration	0.241	<0.001	0.170	0.080
SBP	0.269	<0.001	0.204	0.035
DBP	0.054	0.387	0.180	0.064
FBG	-0.103	0.099	-0.138	0.156
HbA1c	-0.072	0.249	-0.128	0.189
TC	-0.075	0.236	0.007	0.941
TG	-0.182	0.004	0.018	0.855
HDL-C	0.144	0.024	-0.086	0.378
LDL-C	-0.009	0.893	0.000	0.998
Neutrophil count	0.032	0.616	0.002	0.986
Lymphocyte count	-0.202	0.001	-0.285	0.003
NLR	0.152	0.015	0.260	0.007
WBC	-0.061	0.328	-0.099	0.313
PT	0.132	0.112	0.143	0.142
APTT	-0.078	0.347	-0.177	0.067
INR	0.004	0.960	0.096	0.325
Fibrinogen	0.023	0.781	-0.024	0.809
ACR	0.275	<0.001	0.133	0.173
Creatinine	0.049	0.433	0.210	0.030
eGFR	-0.225	<0.001	-0.173	0.075
ABI	-0.130	0.038	-0.201	0.038
VPT	0.191	0.002	0.147	0.021
Hypertension	0.188	0.002	0.126	0.195
CHD	0.205	0.001	0.083	0.394
Stroke	0.128	0.039	0.122	0.210
DFU	0.048	0.438	0.200	0.039
PAD	0.304	<0.001	0.239	0.013
DPN	0.210	0.001	0.136	0.032
DN	0.200	0.001	0.225	0.020
DR	0.074	0.238	-0.041	0.674

**Table 3:** Binary logistic regression analyses of variables contributing to DPN in patients with T2DM.

Variables	Univariate analysis			Multivariate analysis		
	*B*	OR (95% CI)	*P* value	*B*	OR (95% CI)	*P* value
Sex	-0.478	0.620 (0.345-1.114)	0.110			
Age	0.035	1.036 (1.007-1.066)	0.016			
BMI	-0.155	0.856 (0.784-0.935)	0.001			
Diabetes duration	0.043	1.044 (1.003-1.088)	0.037			
SBP	-0.005	0.995 (0.983-1.007)	0.445			
DBP	-0.006	0.994 (0.973-1.017)	0.614			
FBG	0.058	1.059 (1.008-1.113)	0.022			
HbA1c	0.118	1.125 (1.009-1.254)	0.033	0.441	1.554 (1.015-2.378)	0.042
TC	-0.167	0.846 (0.646-1.107)	0.223			
TG	-0.082	0.921 (0.765-1.109)	0.387			
HDL-C	0.474	1.607 (0.685-3.767)	0.275			
LDL-C	-0.163	0.849 (0.604-1.194)	0.348			
Neutrophil count	0.211	1.235 (1.055-1.446)	0.009			
Lymphocyte count	-1.221	0.295 (0.157-0.555)	<0.001			
NLR	0.228	1.257 (1.112-1.419)	<0.001			
WBC count	0.051	1.053 (0.901-1.230)	0.516			
PT	0.182	1.200 (0.827-1.740)	0.337			
APTT	0.055	1.056 (0.986-1.132)	0.120			
INR	0.870	2.387 (0.020-287.730)	0.722			
Fibrinogen	0.404	1.498 (1.108-2.026)	0.009			
BNP	0.020	1.020 (1.006-1.034)	0.004	0.043	1.044 (1.006-1.084)	0.024
ACR	0.000	1.000 (1.000-1.001)	0.112			
Creatinine	0.016	1.016 (1.005-1.026)	0.003			
eGFR	-0.020	0.981 (0.969-0.993)	0.002			
DR	0.820	2.270 (1.060-4.862)	0.035			
DFU	1.570	4.805 (1.597-14.455)	0.005			
PAD	1.153	3.167 (1.571-6.386)	0.001			
CHD	0.534	1.705 (0.818-3.557)	0.155			
Stroke	0.499	1.648 (0.878-3.093)	0.120			

Beta is the standardized coefficient and measures the influence of each variable on DPN; OR is the odds ratio and refers to the risk of DPN.

**Table 4:** Association between quartiles of circulating levels of BNP and the risk of DPN in patients with T2DM.

Circulating BNP (pg/mL)	DPN	
	Odds ratio (95% CI)	*P*
Per SD increase	1.494 (1.130-1.963)	0.004
Quartiles of BNP		
Q1 (0.20–8.00 pg/mL)	1 (reference)	
Q2 (8.01–18.06 pg/mL)	2.900 (1.046-8.038)	0.041
Q3 (18.07–36.34 pg/mL)	5.294 (1.982-14.138)	0.001
Q4 (36.35–100.00 pg/mL)	3.496 (1.277-9.572)	0.015
*P* for trend	0.008	
Q2, Q3, and Q4 versus Q1	3.825 (1.560-9.376)	0.003

Data are expressed as OR (95%CI) + *P* value, unless stated otherwise. OR: odds ratio; CI: confidence interval.

## Data Availability

The data used to support the findings of this study are available from the corresponding author upon request.
